# Configuration Optimization Design of Ti6Al4V Lattice Structure Formed by SLM

**DOI:** 10.3390/ma11101856

**Published:** 2018-09-28

**Authors:** Long Bai, Junfang Zhang, Xiaohong Chen, Changyan Yi, Rui Chen, Zixiang Zhang

**Affiliations:** State Key Laboratory of Mechanical Transmission, Chongqing University, Chongqing 400044, China; 20160702032@cqu.edu.cn (J.Z.); chenxh@cqu.edu.cn (X.C.); 20160713134@cqu.edu.cn (C.Y.); cr@cqu.edu.cn (R.C.); zixiang.randy@outlook.com (Z.Z.)

**Keywords:** lattice structure, selective laser melting, FEA, ground structure method, topology optimization

## Abstract

Previous studies have revealed the influence of various lattice structures on the material density and mechanical properties. However, the majority of the topologies that are considered as study objects directly refer to metal/non-crystal lattice cell configurations. Therefore, this paper proposes a configuration generation approach for generating a lattice structure, which can obtain a lattice configuration that enjoys the advantages of both ultra-low weight and favorable mechanical properties. Based on this approach, a new type of face-centered cubic lattice (all face-centered cubic, AFCC) structure with comprehensively optimal properties in terms of mass and mechanical properties is obtained. The experimental samples are formed with Ti6Al4V by the selective laser melting (SLM) method. Quasi-static uniaxial compression performance experiments and finite element analysis (FEA) are conducted on an AFCC structure and the control group body-centered cubic (BCC) structure. The results demonstrates that our optimized AFCC lattice structure is superior to the BCC structure, with elastic modulus and yield limit increases of 143% and 120%, respectively. For the same degree of deformation, the energy absorbed increases approximately 2.4 times. The AFCC demonstrates significant advantages in terms of its mechanical properties and anti-explosion impact resistance while maintaining favorable ultra-low weight, which validates the hypothesis that the proposed configuration generation approach can provide guidance for the design and further research on ultra-light lattice structures in related fields.

## 1. Introduction

In 2001, Ashby introduced the concept of an ultra-light lattice structure that is defined as a statically/statically indeterminate porous-ordered microstructure that simulates the atomic lattice configuration [1]. Lattice structures have attracted tremendous attention from researchers and engineers for multifunctional design opportunities (i.e., low weight, high strength, high sound absorption, crashworthiness, and thermal properties) as well as their significant potential for a wide range of lightweight applications, e.g., in aerospace, automotive, and biomechanical engineering areas [2,3,4]. Currently, the lightweight and mechanical properties of the lattice structure have become a keystone of study. Ushijima et al. studied the mechanical properties of lattice structures through theoretical modeling, finite element (FE) simulations, and experiments [5].

Since the lattice structure is formed by periodically arranged basic unit cells, the properties of the unit cell thus have an important and even decisive influence on the structural performance [6]. Therefore, this concept has attracted researchers’ interest with studies focused on the lattice structures of different unit cell topologies. Deshpande et al. studied the relative density, effective elastic properties, compressive properties, and shear properties of octahedron lattice structures through theoretical modelling, FE simulations, and experiments [7]. Additionally, the influence of the relative density on mechanical properties was analyzed. Neff and Zheng et al. investigated the effect of the size and number of unit cells on the effective modulus and the impact of the strut element parameters on the mechanical parameters in the diamond lattice, and constructed a theoretical model of the stiffness matrix, tensile stress, yield stress, and shear stress [8,9]. Long Bai et al. proposed the body-centered tetragonal (BCT) unit cell structure, giving full consideration to the relative density, the mechanical properties under a compressive load, and other factors, and successfully obtaining optimized cell parameters exhibiting both low weight and favorable mechanical properties [10]. Maskery et al. deduced the equations of the BCC lattice structure in terms of the relative density, modulus, and ultimate tensile strength based on the empirical formula of Gibson and Ashby, and experimentally studied the effect of the number and size of the BCC unit cells on the mechanical performance [11]. Wallach et al. performed research on the mechanical properties of a triangle-composed three-dimensional (3D) triangle lattice structure and obtained an expression for the stress–strain relationship and Poisson’s ratio of the unit cell structure. The influence of the unit cell size on the elastic modulus, shear modulus, and Poisson’s ratio on the lattice structure was investigated [12]. Kooistra et al. derived an expression for the relative density and ultimate stress in a tetrahedral unit cell. The influence of the relative density on ultimate stress was also analyzed by comparing the results of theoretical modeling and compression experiments [13]. Zheng et al. took the 3D Kagome lattice sandwich structure as their object of research to analyze the equivalent mechanical properties of the Kagome unit cell and study the variation of the capacity to resist impact, because the impact load strength, core geometry, and other factors change (as was found using MSC_Dytran software (MSC_Dytran, Los Angeles, CA, USA)). A proposed optimal solution for resisting the impact was introduced [14]. Mazur et al. analyzed the mechanical properties and deformation failure modes of body-centered cubic (BCC), Face centred cubic unit cell with vertical struts (FCCZ), Face and body centred cubic unit cell with vertical struts (FBCCZ), Face and body centred cubic unit cell with vertical struts and no end webs (FBCCZO) and Face and body centred cubic unit cell with vertical and horizontal struts (FBCCXYZ) lattice structures that shared the same unit cell size using experiments and numerical analysis [15].

The previous studies mentioned above investigated the influencing factors of the characterization model and mechanical properties in terms of the lattice structure based on a unit cell. Nevertheless, these unit cell topologies of the lattice structure are generally obtained by simulating or referring to metal lattice patterns or non-metallic crystal cells, such as octahedral, diamond, or BCC, rather than by generating the lattice by theoretical modeling. Considering the lattice structure concept and the existing features of its configuration, the lattice structure is constructed by certain special nodes in the cell body (e.g., the vertex, face center, and body center) connected using struts, corresponding to a truss-type structure. This truss-like discrete structure is also the research object of structural topology optimization at the initial stage [16].

Therefore, we propose a generating method that uses discrete structure topology optimization theory to obtain the optimized unit cell configuration of the lattice structure. First, the ground structure design model of the lattice unit cell is established using the ground structure method; then, by taking into account the lightweight and high-strength properties of the lattice structure such as the relative density of the material and the mechanical properties under compressive load, a topology optimization mathematical model of the unit cell is constructed. The firefly optimization strategy algorithm is introduced to solve the model to obtain the optimal configuration and size of the unit cell. Last, the method of selective laser melting (SLM) is adopted to manufacture the experimental samples of the optimized lattice structure and the experimental samples of the typical BCC structure using the material Ti6Al4V. The compression performance experiment, finite element analysis (FEA), and comparative analysis are conducted to demonstrate the feasibility and correctness of the proposed optimization design method and the obtained results.

## 2. Model Establishment

### 2.1. Mathematical Model

#### 2.1.1. Establishment of the Ground Structure

The ground structure is a set of nodes constructed by boundary nodes, load nodes, and structural nodes, with any two arbitrary nodes connected by discrete strut elements [17]. The ground structure method refers to a generating target topology structure approach that can select the minimum cross-section area of each strut, or deletes unnecessary struts and nodes using an optimization algorithm operated on an initial structure that contains all of the possible nodes and struts formed by connecting each two nodes [18]. The ground structure method as a discrete structure topology generation method is a commonly employed topology generation method. This method offers many advantages when combined with modern intelligent bionic algorithms. For instance, this approach can simultaneously simplify or enhance the strut structure and can consider the coupling relationship between the cross-sectional variables and topological variables.

**Figure 1:**
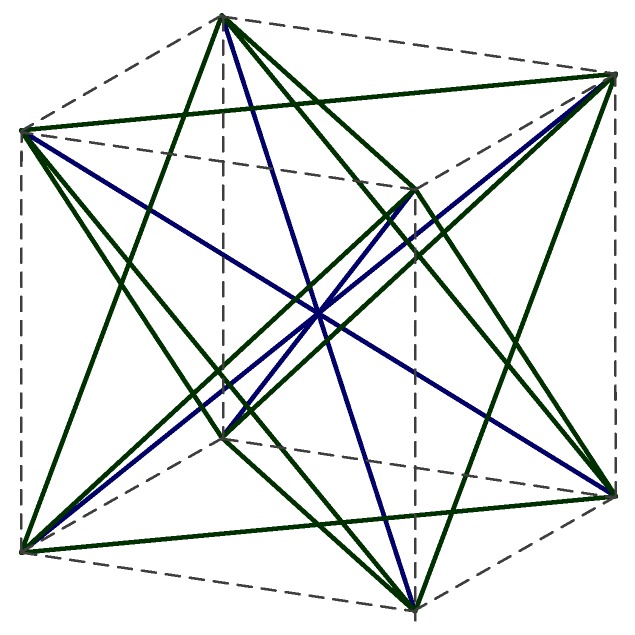
Ground structure schematic.

According to the definition of the ground structure, this work chooses the eight vertices—six face-centered and one body-centered—to form a node set. In a porous foam structure, the elastic modulus of the model will scale linearly with the relative density if a straight strut is used throughout the entire model, thus limiting the deformation type to longitudinal stretching and compression only. As a result, the majority of porous foam does not contain horizontal and vertical struts [19], which can allow beam bending to play a role. Therefore, a ground structure that excludes the horizontal and vertical struts, as shown in Figure 1, a strut element is formed between two adjacent nodes. According to the spatial position, strut elements are divided into two groups: face struts and body heart struts. The face struts function as a connection between the vertices and face centers. The heart struts connect the vertices and the top of the body center. The number of strut elements that connect the 15 nodes (excluding the vertical and horizontal struts) is 32.

#### 2.1.2. Solution for the Axial Force *F_N_*

Reference made the following assumptions for the lattice structure [5]:

- All of the struts in the unit cells are homogeneous with circular cross-sections.
- The material is isotropic, and the compressive and tensile stress–strain relationships are equivalent for all of the strut elements.
- The strut element experiences only axial tension, compression, and bending; the effects of torsion are negligible.

Based on the above assumptions, with reference to Euler–Bernoulli beam theory [20], the strut elements can be considered Euler–Bernoulli beams.

1. Establishment of Coordinate System

**Figure 2:**
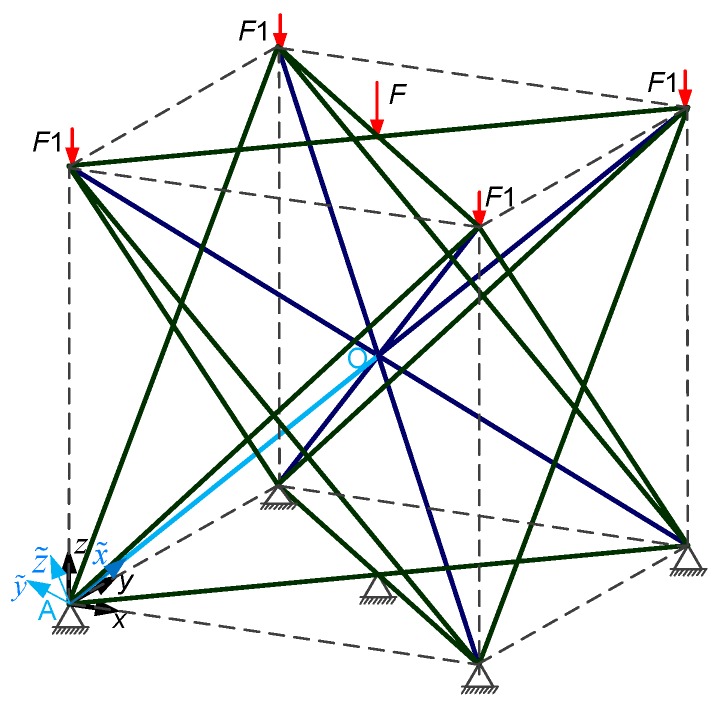
Force analysis diagram of the ground structure under applied loads.

The *x*, *y*, and *z* coordinates in Figure 2 comprises the global coordinate system of the structure, in which ${\tilde{x}}_{i}$, ${\tilde{y}}_{i}$, ${\tilde{z}}_{i}$ is the local coordinate system of the *i*-th element (*i* = 1, 2, …, 32), and ${\tilde{x}}_{i}$ is the axis of the element. The direction of ${\tilde{x}}_{i}$ for the body center element is the vertex to the body center, and the direction of ${\tilde{x}}_{i}$ for the face center element is vertex to the face center. Let the vector *P_i_* (*i* = 1, 2, …, 32) be parallel to the z-axis and pass through the end of *i*-th element, then ${\tilde{y}}_{i}=m\left(P_{i}\times {\tilde{x}}_{i}\right)$ *m* > 0, and ${\tilde{z}}_{i}=\left({\tilde{x}}_{i}\times {\tilde{y}}_{i}\right)$. As shown in Figure 2, the local coordinate system $\tilde{x}$, $\tilde{y}$, $\tilde{z}$ of the OA strut element. In the lattice structure, one vertex is shared by four unit cells, and one face center is only one unit cell. The applied load *F* is four times greater than *F*_1_.

2. Structural Stiffness Matrix

Based on the above assumptions, the relationship between the strain and the displacement, as well as the generalized Hooke’s law, the element stiffness matrix in the global coordinate system is obtained:

(1) $$K^{e}=T^{T}{\tilde{K}}^{e}T$$

where ${\tilde{K}}^{e}$ is the element stiffness matrix in the local coordinate system, and *T* is the coordinate transformation matrix from the global coordinate system to the local coordinate system.

$${\tilde{K}}^{e}=\left[\begin{array}{cc} k_{1} & k_{2}\\ k_{3} & k_{4} \end{array}\right]$$

where

$$k_{1}=\left[\begin{array}{ccc} \frac{EA}{l} & 0 & 0\\ 0 & \frac{12EI}{l^{3}} & \frac{6EI}{l^{2}}\\ 0 & \frac{6EI}{l^{2}} & \frac{4EI}{l} \end{array}\right];k_{2}=\left[\begin{array}{ccc} -\frac{EA}{l} & 0 & 0\\ 0 & -\frac{12EI}{l^{3}} & \frac{6EI}{l^{2}}\\ 0 & -\frac{6EI}{l^{2}} & \frac{2EI}{l} \end{array}\right];\;k_{3}=\left[\begin{array}{ccc} -\frac{EA}{l} & 0 & 0\\ 0 & -\frac{12EI}{l^{3}} & -\frac{6EI}{l^{2}}\\ 0 & \frac{6EI}{l^{2}} & \frac{2EI}{l} \end{array}\right];k_{4}=\left[\begin{array}{ccc} \frac{EA}{l} & 0 & 0\\ 0 & \frac{12EI}{l^{3}} & -\frac{6EI}{l^{2}}\\ 0 & -\frac{6EI}{l^{2}} & \frac{4EI}{l} \end{array}\right];$$

where *E* is the elastic modulus of the raw material; *A* and *l* are the strut element cross-sectional area and strut length, respectively; and *I* is the sectional moment of inertia.

$$T=\left[\begin{array}{ccc} l_{x} & m_{x} & n_{x}\\ -\frac{m_{x}}{\sqrt{1-n_{x}^{2}}} & -\frac{l_{x}}{\sqrt{1-n_{x}^{2}}} & 0\\ -\frac{l_{x}n_{x}}{\sqrt{1-n_{x}^{2}}} & -\frac{m_{x}n_{x}}{\sqrt{1-n_{x}^{2}}} & \sqrt{1-n_{x}^{2}} \end{array}\right]$$

where *l_x_*, *m_x_*, and *n_x_* are the direction cosines of the angles between the ${\tilde{x}}_{i}$ (*i* = 1, 2, …, 32) axis of the local coordinate system, and the *x*, *y*, and *z*-axis of the global coordinate system, respectively.

According to the deformation coordination condition and combining the element stiffness matrix, the structural stiffness matrix *K* can be obtained in the global coordinate system.

3. Solution for the Axial Force *F_N_*

By the matrix displacement method, the relationship between the nodes load matrix and the displacement matrix is obtained as:

(2) P=K•Δ

where Δ is the displacement matrix of all of the nodes in the global coordinate system, and *P* is the structural nodes load matrix, as determined by the applied load.

Extract the strut end displacement matrix $\Delta^{e}$ from the nodal displacement matrix Δ according to ${\tilde{\Delta }}^{e}=\left[\begin{array}{cc} T & \\ & T \end{array}\right]\Delta^{e}$, which is the strut end displacement matrix ${\tilde{\Delta }}^{e}$ in the local coordinate system. Similarly, the equivalent node load matrix ${\tilde{P}}^{e}$ in the local coordinate system can be obtained from the structural node load matrix *P* in the global coordinate system.

According to the principle of force balance, the internal force at the end of the element strut is:

(3) $${\tilde{F}}^{e}={\tilde{K}}^{e}{\tilde{\Delta }}^{e}-{\tilde{P}}^{e}$$

where ${\tilde{F}}^{e}$ is the element-end internal force matrix in the local coordinate system, and the first element extracted from ${\tilde{F}}^{e}$ is the axial force *F_N_*.

## 3. Model Optimization

This study aims to obtain a new lattice structure that exhibits both low weight and superior mechanical properties using topology optimization based on the proposed ground structure. Given a material object, the mass depends on its volume. The volume of the lattice structure is the sum of the volumes of the struts. The cross-sectional area and strut length are the parameters influencing the strut volume. The length of each strut element is determined after the sizes of the unit cell structure are known. Here, we treat the cross-sectional area of each strut as a design variable adjusted to obtain the minimum volume. A mathematical model is constructed with regards to the constraints of the applied forces.

### 3.1. Design Variables

We take the cross-sectional areas of 32 strut elements in the ground structure as the design variables:

(4) $$\vec{S}=(S_{1},S_{2},\ldots ,S_{i},\ldots ,S_{32})$$

where *s_i_* is the cross-sectional area of the *i*-th strut element.

### 3.2. Objective Function

The ground structure method can transfer the topology optimization problem to the cross-sectional area optimization problem [18]. Thus, the goal of optimization in this article is to find cross-sectional vectors for the case of the minimum volume satisfying the constraints. The objective function is as follows:

(5) $$f\left(\vec{S}\right)=\min\left(\sum_{i=1}^{32}s_{i}l_{i}\right)$$

where *l_i_* is the strut length of the *i*-th strut element.

### 3.3. Constraints

With an applied load, the axial force of each strut element must be less than the product of the allowed stress of the chosen material and the cross-sectional area of each strut element. Since the volume of each strut element is non-negative, the constraints apply to the cross-sectional areas of the strut element.

#### 3.3.1. Force Constraints

To ensure sufficient strength for the structure, the actual stress of the strut element should be lower than the ultimate stress with applied load. In strength calculations, the ultimate stress is divided by a factor greater than one, and the result obtained is referred to as the allowable stress. The allowable stress is the ceiling of the strut element working stress, which requires the work stress to not exceed the allowable stress.

(6) $$\sigma =\frac{\left|F_{N}\right|}{A_{\mathrm{OA}}}\leq \left[\sigma \right]$$

where σ is the working stress, *A*_OA_ is the strut element cross-sectional area, and [σ] is the allowable stress.

From Equation (6), we obtain:

(7) $$\left|F_{N}\right|\leq A_{\mathrm{OA}}\left[\sigma \right]$$

This is bound by the force as follows:

(8) $$g_{i}\left(\vec{S}\right)={\left|F_{N}\right|}_{i}\leq s_{i}\left[\sigma \right](i=1,2,\ldots ,32)$$

where ${\left|F_{N}\right|}_{i}$ is the absolute value of the axial force of the *i*-th strut element.

#### 3.3.2. Cross-Sectional Area Constraints

Since the volume is non-negative and the absolute value of the axial force in Equation (7) cannot be less than zero, the area should not be less than zero. Therefore, the area constraints are given as:

(9) $$s_{i}\geq 0,(i=1,2,\ldots ,32)$$

### 3.4. Solution Process Based on the Firefly Algorithm

The firefly algorithm is a convenient, flexible, and highly universality random search algorithm that can achieve the optimization through mutual attraction between the firefly individuals. The firefly algorithm is a random optimization algorithm based on group search, which belongs to the group intelligent optimization algorithm class [21]. The algorithm first initializes a set of solutions randomly, and then continuously updates these solutions during the iterations until the optimal value of the problem is found. Relative to other intelligent optimization algorithms, the firefly algorithm enjoys the advantages of a simpler concept, clearer process, fewer parameters to be adjusted, easier implementation, high searching speed and precision; thus, it is a feasible and effective optimization method.

#### 3.4.1. Solution of the Optimization Model

**Figure 3:**
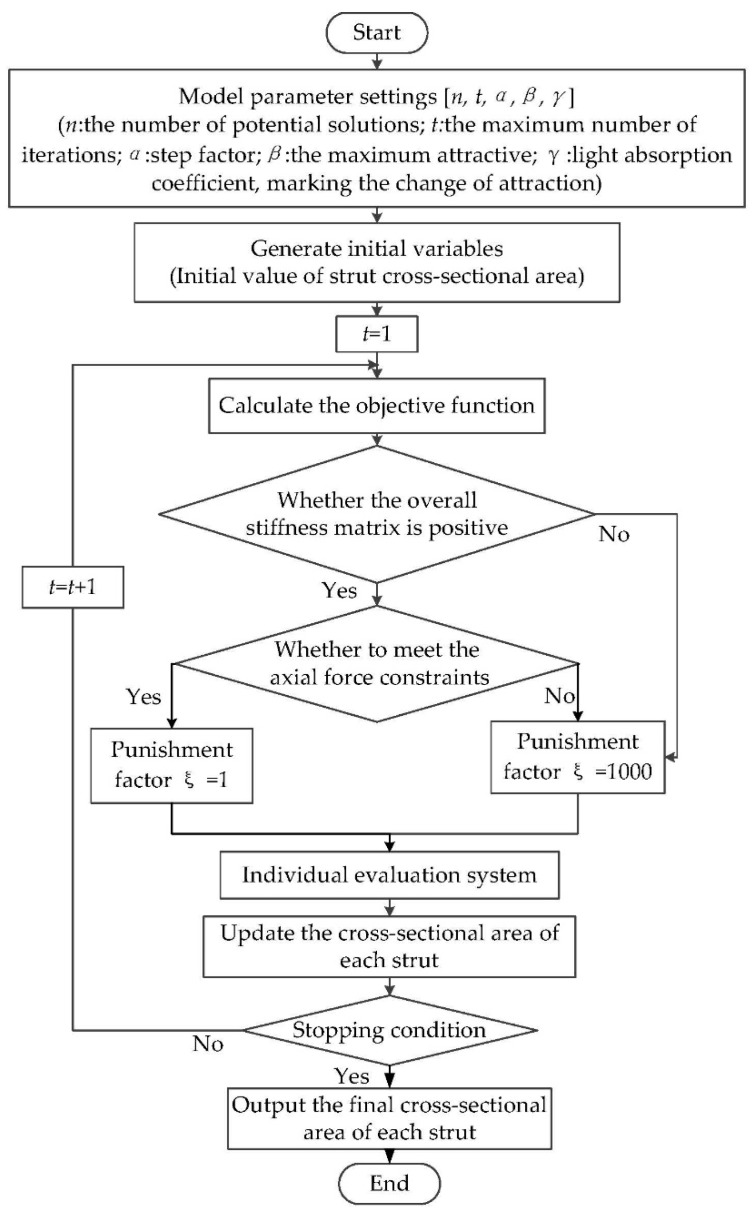
Topology optimization flow chart based on the firefly algorithm.

Using the firefly algorithm combined with the models described above, the discrete variable strut structure topology optimization process is shown in Figure 3. In this process, punishment factor ξ is used to determine whether the axial force meets the constraint condition. If meeting the constraint condition ξ = 1, otherwise ξ = 1000.

#### 3.4.2. Establishment of the Optimization Mathematical Model

According to the constraints of Equations (8) and (9), combined with the objective function, an evaluation function can be formed from the constraints and the objective function. The topologically optimized mathematic model is given by:

(10) $$\{\begin{array}{c} find\begin{array}{c} \vec{S}=(s_{1},s_{2},\ldots ,s_{i},\ldots ,s_{32}) \end{array}\\ \min\begin{array}{c} f(\vec{S}) \end{array}\\ s.t.\;g_{i}\left(\vec{S}\right)\leq s_{i}\left[\sigma \right](i=1,2,\ldots ,32)\\ \vec{S}\geq 0 \end{array}$$

### 3.5. Optimization Results

**Figure 4:**
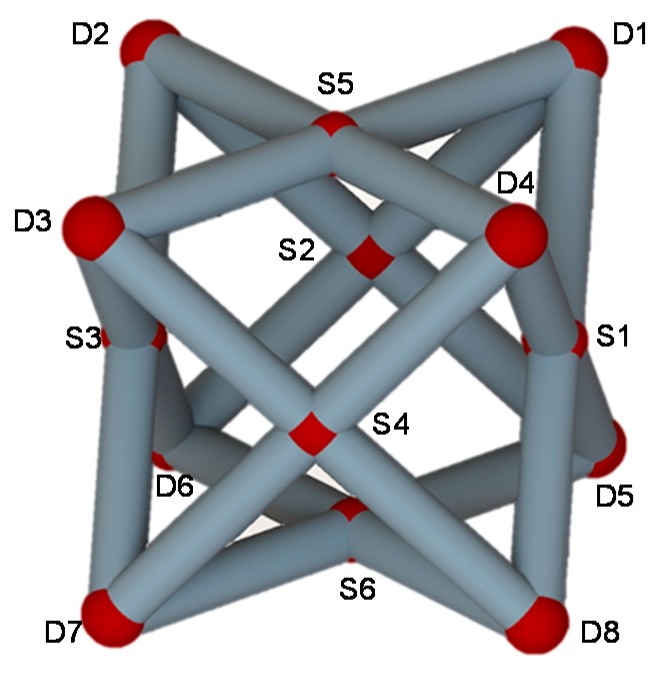
Generated optimal unit cell model based on the topology optimization method.

For the ground structure that is subjected to the applied loads in Figure 2, the mathematical model and flow chart of the strut structure topology optimization were established in the previous sections. MATLAB software (MATLAB R2016a, Natick, MA, USA) is used to solve the model. The new unit cell structure from the ground structure is based on the topology optimization method, as shown in Figure 4, and is an all face-centered cubic (AFCC) structure, in which the AFCC structure node consist of eight vertices *D_i_* (*i* = 1, 2, …, 8) and six face-centered *S_i_* (*i* = 1, 2, …, 6). Table 1 lists the specific optimization results of the cross-sectional areas in one strut element; the cross-sectional area is taken after the decimal point. B represents the body-centered point. A strut element is formed by two adjacent nodes. For example, D7B represents an element composed of vertex D7 and body-centered point B. Since the position update formula in the firefly algorithm contains random items of specific coefficients, the difference in the cross-sectional area between the individual strut elements is caused.

The obtained optimization results are subsequently processed. For the convenience of modeling, the optimized radius results are rounded to one decimal and listed in Table 2.

The optimization results of the 32-strut element radii are presented in Table 2. The radius value that is optimally calculated by the body-centered strut element is 0 mm, and the radius value of all four side planes is 0.4 mm. To ensure the symmetrical distribution of the unit cell structure, the radii of the strut elements on the ground (D_5_ D_6_ D_7_ D_8_) are set to 0.4 mm in the experiment.

## 4. FEA

FEA has high precision, low cost, and a short period, which is a common method for studying metal lattice structures. This section uses ABAQUS for finite element simulation, simulates quasi-static compression experiments of lattice structures, and predicts the mechanical properties of lattice structures.

### 4.1. FEA Model

The BCC lattice structure has the characteristics of simple topology and isotropy, which render it well-suited to the SLM-forming process, which is the most common lattice and has important practical application value [5,22]. Therefore, this paper selects the isotropic BCC lattice structure as a reference for the performance comparative analysis of the optimized isotropic AFCC structure. Relative density is an important indicator to describe the ultra-light property in lattice material, and is one of the key factors that influences the mechanical properties. To compare two types of lattice structures under the same conditions, the controlling variable method is applied, requiring both structure configurations to have the same relative density.

**Figure 5:**
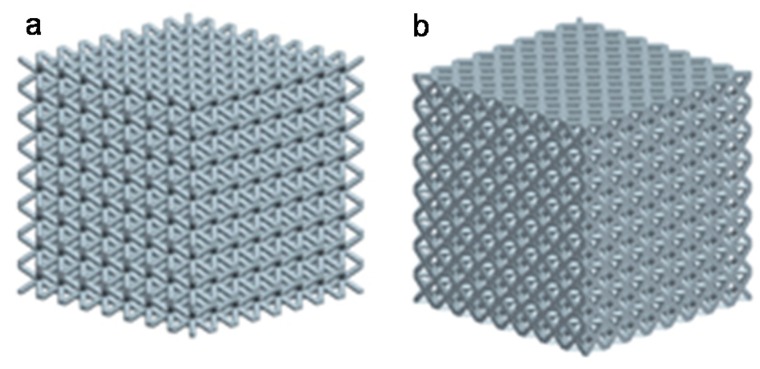
Schematic diagram of three-dimensional (3D) model of finite element analysis (FEA): (**a**) BCC; (**b**) AFCC.

The 3D model of the AFCC and BCC lattice structure in this simulation is established in Pro/Engineer. The model parameters are shown in Table 3, and the three-dimensional model is shown in Figure 5.

### 4.2. FEA Results

In order to simulate the quasi-static compression experiments of lattice structures, the dynamic explicit analysis in ABAQUS is used. The damage mode is simulated by Johnson–Cook damage model efor quasi-static compression experiments. In consideration of no literature about the Johnson–Cook model, parameters of Ti6Al4V were found by SLM. By referencing the research of Zhang et al. [23], this article selects the Johnson–Cook constitutive model parameters and Johnson–Cook fracture model parameters of hot rolled Ti6Al4V instead of SLM, which are assigned in the material property manager. The difference in the forming process of the sample may result in performance differences, but it does not affect the approximate prediction of the mechanical properties of the simulation results. The quasi-static compression simulation of lattice structures by ABAQUS was conducted, which predicts the mechanical properties of the structure before sample experiments, but not instead of sample experiments.

**Figure 6:**
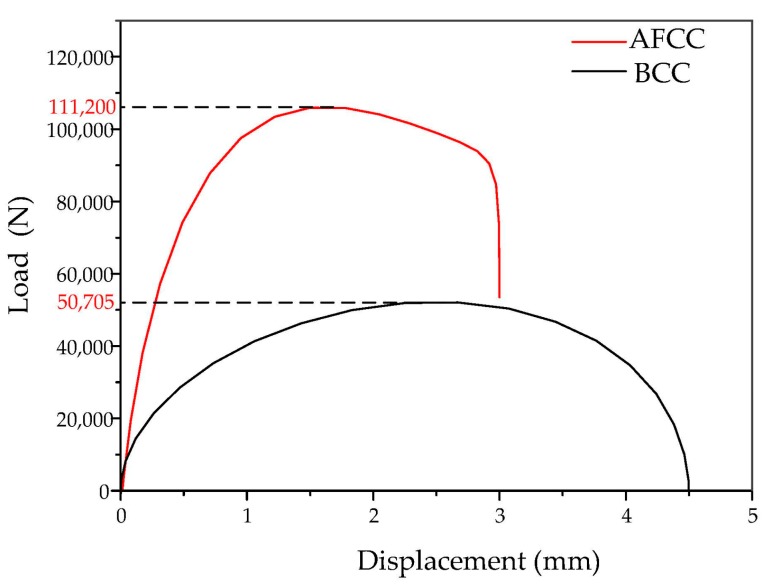
Load–displacement curves of AFCC and BCC lattice structures.

**Figure 7:**
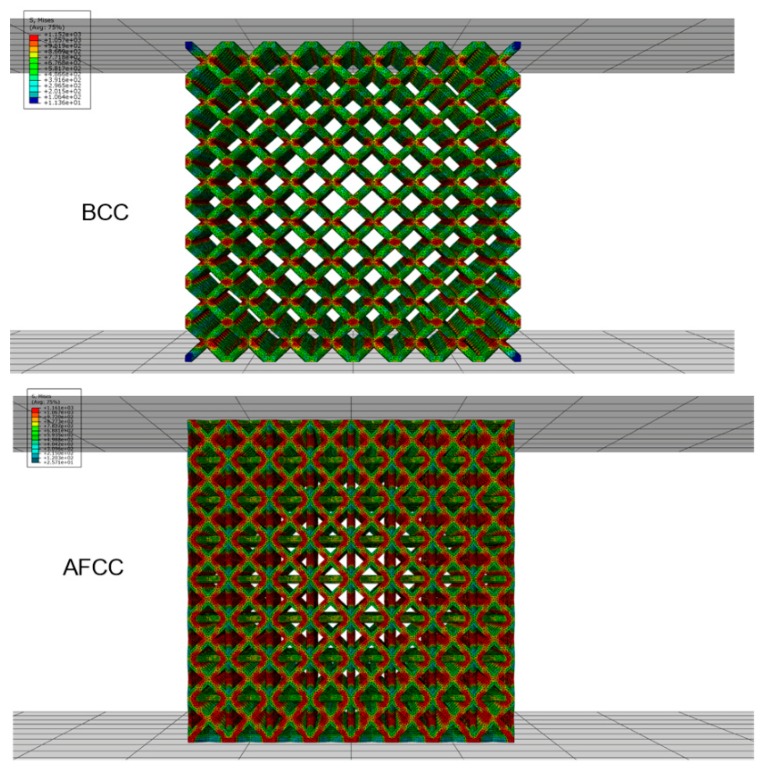
Stress nephogram of AFCC and BCC lattice structure.

**Figure 8:**
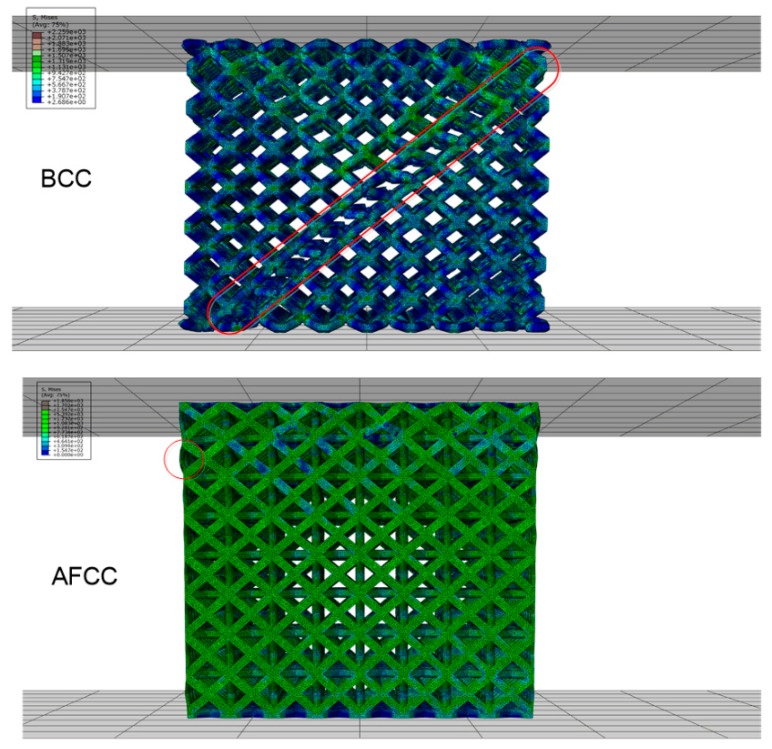
Failure diagram of AFCC and BCC lattice structures.

From the FEA results, the load and displacement curves of the lattice structure (Figure 6), the stress nephogram (Figure 7) and the failure diagram (Figure 8) can be obtained. It can be seen from the load and displacement curves of the lattice structure that the deformation process of the lattice structure can be divided into the elastic stage, the hardening stage, and the failure stage. The maximum loads that the AFCC structure and the BCC structure can withstand are 111,200 N and 50,705 N, respectively. It can be seen from the stress nephogram that the stress of both structures is concentrated at the node, and the stress near the midpoint of the strut element is the smallest. It can be seen from the failure diagram of the lattice structure that the BCC structure has fracture failure along the 45° direction, while the AFCC structure has yield failure.

## 5. Experimental Study

In this study, the experiment samples of the BCC reference group and the AFCC optimized structure are manufactured using the Ti6Al4V material and the SLM forming process. Additionally, the quasi-static uniaxial compression test is conducted using a universal material testing machine.

### 5.1. Manufacturing of Sample and Experiment Conditions

**Figure 9:**
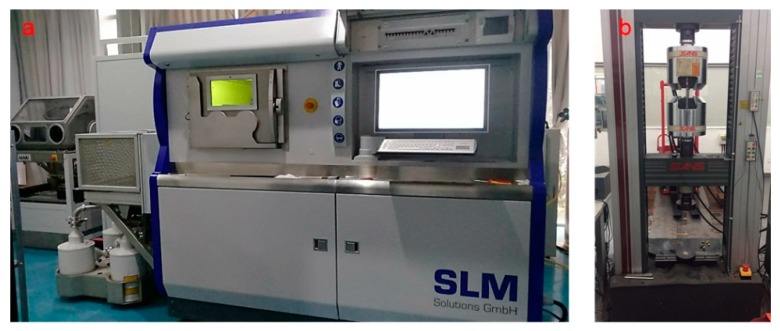
Experimental apparatus: (**a**) SLM500HL laser melting rapid prototyping machine; (**b**) universal material testing machine.

The forming equipment is the SLM rapid prototyping machine (SLM500HL, SLM Solutions Group AG, Lübeck, Germany), as shown in Figure 9a. The machine parameters are presented in Table 4. The raw material is the Ti6Al4V titanium alloy powder, which is molded using the line scanning method.

To characterize the mechanical properties of the experimental sample, the quasi-static uniaxial compression test is performed using a universal material testing machine (CMT5205, Shenzhen Wance Testing Machine Co., Ltd., Shenzhen, China) (Figure 9b). At room temperature, a single sample is placed on a horizontal table of the test machine. The machine indenter compresses the sample at the speed of 1 mm/min, and the load–displacement data are recorded. When plastic deformation or destructive failure occurs, the load decreases rapidly in the load–displacement curve, at which point the experiment terminates.

**Figure 10:**
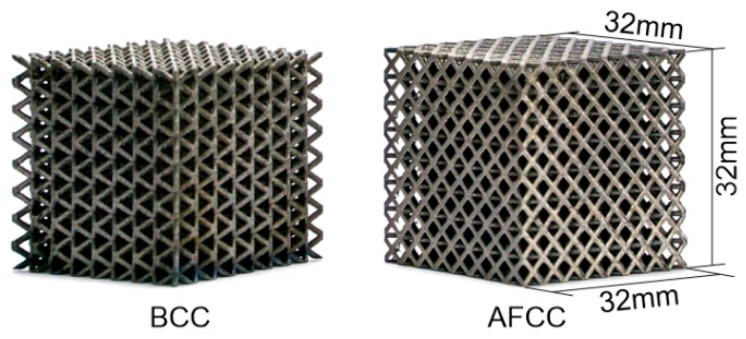
Selective laser melting (SLM) forming sample of AFCC and BCC structures with dimensions of 32 mm × 32 mm × 32 mm.

According to the data presented in Table 3, the lattice structure sample is constructed according to the periodic arrangement using the SLM forming process. The manufactured BCC and AFCC samples are shown in Figure 10.

### 5.2. Experimental Results

We performed the compression test for the two types of structures under the stated conditions. Based on the test results, comparative analysis was conducted separately in terms of the mechanical properties, failure modes, and energy absorption.

### 5.3. Destructive Failure Model

The destructive failure graph shows that the AFCC optimized structure and the BCC reference structure exhibit overall damage along the direction at 45°, and that local deformation, double shear slip, and localized failure all appear. These results are consistent with previous studies regarding the cracking mechanism of the Ti6Al4V material based on the SLM formation. The phenomena of the local damage [10,24], fracture at the nodes, and other points in the lattice structure observed in the uniaxial compression test were attributed to the residual stress produced by high-temperature gradients, powder particles that adhered to the surface of the strut, and other factors.

**Figure 11:**
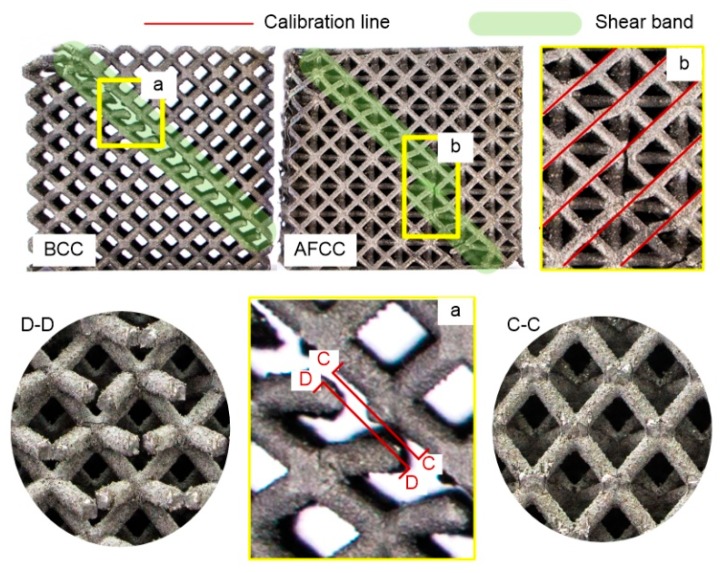
Sample failure graph of AFCC and BCC lattice structures: (a, C–C and D–D) partial enlargement of BCC structural failure; (b) partial enlargement of AFCC structural failure.

The BCC reference structure fractures along the global 45° direction are divided into two parts, as shown in Figure 11 (a, C-C and D-D). The graph shows that the strut of the damaged unit cell fractures and disengages from the node with a flattened cleft, which is classified as a brittle fracture. The fracture plane is the plane where a set of nodes is observed with a neat fracture surface, indicating that the node at a 45° inclined plane undergoes the maximum stress. Conversely, the AFCC optimized structure does not experience global damage, and only plastic deformation failure occurs along the 45° direction in the strut element. As shown in Figure 11b, the orientation line slides along the 45° direction.

In the material compression test, a typical stress–strain curve can be categorized into four stages [25]:

(1) The process when the stress reaches its peak.
(2) The stress decreases with a shear slip band along the 45° direction.
(3) The stress increases to a relatively small peak, and the fracture occurs along the 45° direction.
(4) The occurrence of global structural damage.

Fracture damage to the structure is the most dangerous type of failure. To avoid structural fracture, the discovery of the structural slip phenomenon plays a crucial role. Experimental results show that the failure of the AFCC optimized structure occurs at the second stage at a time when global damage has appeared in the reference BCC structure. In conclusion, for the same manufacturing and size constraint conditions, the AFCC configuration obtained in this study shows better performance in terms of preventing global damage.

#### 5.3.1. Mechanical Performance

**Figure 12:**
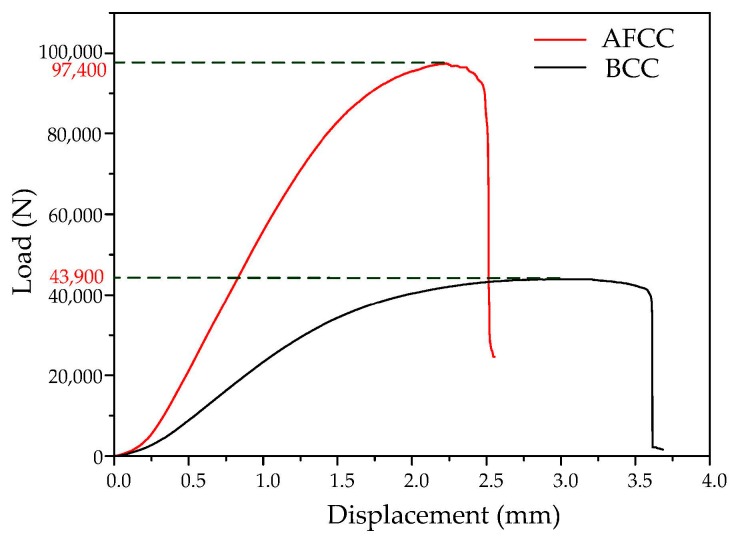
Experimental load–displacement curves of AFCC and BCC lattice structures.

Using a universal material testing machine, a quasi-static uniaxial compression test was conducted. In the compression process, the relationship between the applied load *F*, the time *t*, and the sample deformation δ = *vt* can be measured. The experimental load and displacement curves are shown in Figure 12. The maximum load of the AFCC is 97,400 N, while the maximum load of the BCC is 43,900 N. Compared with BCC, the maximum load of the AFCC is about twice as large.

**Figure 13:**
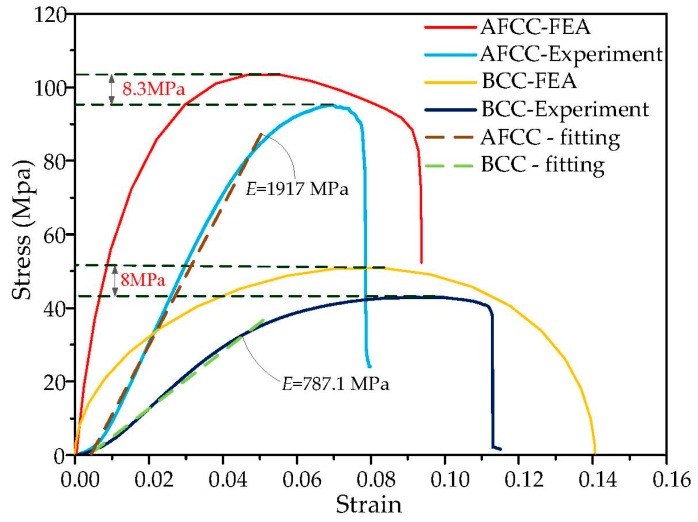
FEA and experimental stress–strain curves and fitting curves of AFCC and BCC lattice structures.

The nominal stress *σ*_nom_ of the lattice structure is calculated by dividing the externally applied load by the sample cross-sectional area W. The nominal strain *ε*_nom_ is obtained by dividing the deformation δ by the initial height *h* of the lattice structural mode. Therefore, the experimental and FEA stress–strain curves comparison of the AFCC optimized structure and BCC reference that are shown in Figure 13 can be obtained.

For an accurate comparison of the elastic moduli of the two structures, linear fitting is applied to the linear elastic stage of the stress–strain curve using MATLAB software. The obtained fitting curve is shown by the dotted line. The fitting result shows that the elastic modulus values for BCC and AFCC are approximately 787.1 MPa and 1917 MPa, respectively. The latter value is approximately 2.44 times larger than the former value. In the compression experiment, the interaction of the beam-column reduces the elastic modulus, whereas the elastic modulus is increased in the stretching process. Consequently, the linear elastic part in the stress–strain curve is not completely linear, but exhibits a concave downward form [26]. There is an error between the elastic stage experiment and the FEA, but the slope of the elastic stage obtained by the finite element simulation is similar to the slope of the fitting curve.

The measured limit stress of AFCC is 95.2 MPa, which is approximately 2.2 times larger than the measured limit stress of BCC, which is 42.87 MPa. For the ultimate stress analysis of AFCC and BCC, the FEA results are 8.3 MPa and 8 MPa higher than the experimental results, respectively. This is due to the machining error, the dimension error of the samples, the experimental conditions, and the different forming process. So, the error between FEA and the experiment is acceptable.

#### 5.3.2. Energy Absorption Performance

Relative to conventional materials, the metal 3D lattice material offers the advantages of low weight, high strength, the ability to resist explosion and impact, efficiency for releasing and insulating heat, the capacity to absorb electromagnetic waves and sounds, and other aspects [27]. Among these, the ability to resist explosion and impact corresponds strongly to the structure energy density.

**Figure 14:**
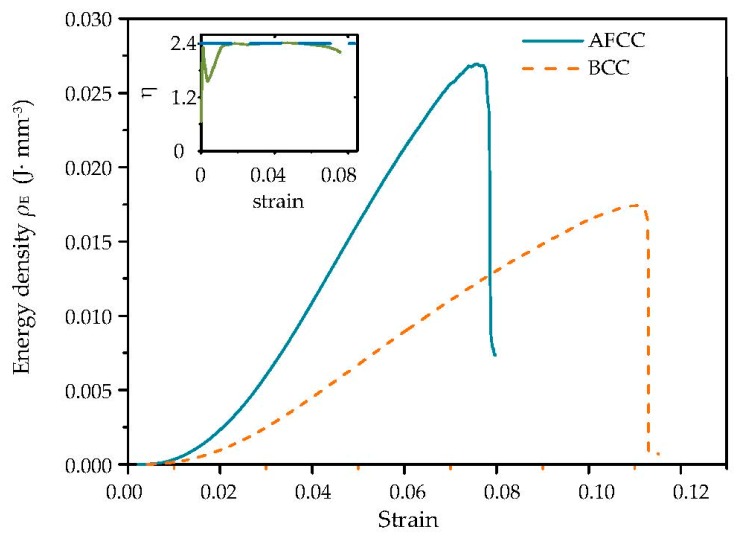
Energy analysis curves of AFCC and BCC lattice structures; the inset shows the ratio of the AFCC and BCC structures energy densities.

According to the test results, the energy analysis plots (Figure 14) of the AFCC and BCC structures were obtained. The vertical axis represents the energy density of the lattice structure, which is expressed as:

(11) $$\rho_{E}=\frac{F\delta }{V}$$

where *F* is the externally applied load (N) and *V* is the volume of the lattice sample (mm^3^). The decreasing part of the curve implies that the structure has failed. The energy density is proportional to the strain in the two lattice structures before failure, and the energy density of the AFCC structure is considerably larger than that of the BCC under the same strain, indicating that the deformation of the AFCC structure is much smaller than that of the BCC under the same impact. The curve of the ratio of the two structures’ energy densities (*η* = *ρ*_E-AFCC_/*ρ*_E-BCC_) and the strain shows that the energy density of the AFCC structure reaches a value that is approximately 2.4 times that of the BCC structure at the elastic deformation stage in the compression process.

## 6. Conclusions

Unlike studies in which molecule-simulating structures are directly employed to conduct a lattice structure design, this study constructs a lattice unit cell ground structure that consists of a finite number of strut elements based on a discrete structure topology optimization method. A lattice configuration unit cell optimization model is established with the cross-section areas of each strut element in the ground structure. The cross-sections are taken as the design variables, the axial applied force is taken as the constraint function, and the minimum volume is taken as the evaluation function. Then, the firefly algorithm is employed as an optimization strategy to obtain the optimal size of each strut element of the confirmed general unit cell ground structure. The strategy generates the optimal new lattice structure (AFCC), which provides a theoretical reference for the design of other lattice material configurations.

This work also uses a typical BCC lattice structure as a reference, with the manufacturing, tests, and FEA performed for both the BCC reference sample and the optimized AFCC sample with the same density parameters and under the same experimental and FEA conditions. Experimental and FEA results show that the AFCC configuration outperforms the BCC configuration in terms of avoiding global damage. The elastic modulus and yield limit are increased by 144% and 122%, respectively, under the same lightweight condition, which emphasizes the prominent advantage in mechanical properties; for the same deformation, the amount of energy absorbed by the AFCC structure is 2.4 times larger than for the BCC structure, indicating a better ability to resist explosion and impact.

FEA provides effective evidence of the experimental results, while also reducing a great deal of time and money for the study of the lattice structure. The proposed optimization model also applies to other lattice structure materials. In practical engineering applications, with specific material properties and manufacturing processes, the optimized lattice configuration and its size can be obtained by setting the corresponding constraints. These parameters are crucial for determining the mechanical properties of Ti6Al4V by SLM in FEA model. Next, we will further study the effect of the SLM forming process on the failure performance of Ti6Al4V, and realize the perfect prediction of the simulation as soon as possible.

## Figures and Tables

**Table 1:** Ground structure optimization results of the cross-sectional areas of each strut element.

Element	A (mm^2^)	Element	A (mm^2^)	Element	A (mm^2^)	Element	A (mm^2^)
D3S5	0.5	D8S1	0.5	D7S3	0.5	D3B	0
D4S5	0.4	D5S1	0.6	D6S3	0.5	D4B	0
D1S5	0.4	D1S1	0.5	D2S3	0.5	D1B	0
D2S5	0.4	D4S1	0.4	D3S3	0.5	D2B	0
D7S4	0.5	D7S6	0	D5S2	0.4	D7B	0
D8S4	0.6	D8S6	0	D1S2	0.5	D8B	0
D4S4	0.5	D5S6	0	D2S2	0.4	D5B	0
D3S4	0.5	D6S6	0	D6S2	0.5	D6B	0

**Table 2:** Strut element radius represents the optimization result of the ground structure.

Element	R (mm)	Element	R (mm)	Element	R (mm)	Element	R (mm)
D3S5	0.4	D8S1	0.4	D7S3	0.4	D3B	0
D4S5	0.4	D5S1	0.4	D6S3	0.4	D4B	0
D1S5	0.4	D1S1	0.4	D2S3	0.4	D1B	0
D2S5	0.4	D4S1	0.4	D3S3	0.4	D2B	0
D7S4	0.4	D7S6	0	D5S2	0.4	D7B	0
D8S4	0.4	D8S6	0	D1S2	0.4	D8B	0
D4S4	0.4	D5S6	0	D2S2	0.4	D5B	0
D3S4	0.4	D6S6	0	D6S2	0.4	D6B	0

**Table 3:** Model parameters. AFCC: all face-centered cubic, BCC: body-centered cubic.

Model	Model Size Length × Width × Height (mm^3^)	Unit Cell Number Length × Width × Height	Relative Density	Cell Strut Radius R (mm)
BCC	32 × 32 × 32	8 × 8 × 8	0.26	0.44
AFCC	32 × 32 × 32	8 × 8 × 8	0.26	0.4

**Table 4:** Primary SLM500HL parameters.

Actual Power (W)	Scanning Interval (mm)	Layer Thickness (μm)	Density (%)
275	0.12	30	99.5
